# Assessing the impact of COmorbidities and Sociodemographic factors on Multiorgan Injury following COVID-19: rationale and protocol design of COSMIC, a UK multicentre observational study of COVID-negative controls

**DOI:** 10.1136/bmjopen-2024-089508

**Published:** 2025-03-06

**Authors:** Simran Shergill, Mohamed Elshibly, Sandeep S Hothi, Kelly S Parke, Rachel J England, Joanne V Wormleighton, George J Hudson, Elizabeth M Tunnicliffe, James Wild, Stephen M Smith, Sue Francis, Mark Toshner, Naveed Sattar, Kamlesh Khunti, Christopher E Brightling, Charalambos Antoniades, Colin Berry, John P Greenwood, Alastair Moss, Stefan Neubauer, Gerry P McCann, Betty Raman, Jayanth Ranjit Arnold

**Affiliations:** 1 Department of Cardiovascular Sciences and the National Institute for Health Research Leicester Biomedical Research Centre, Glenfield Hospital, University of Leicester, Leicester, UK; 2 Department of Cardiology, Heart and Lung Centre, Royal Wolverhampton NHS Trust, Wolverhampton, UK; 3 Institute of Cardiovascular Sciences, University of Birmingham, Birmingham, UK; 4 Department of Radiology, University Hospitals of Leicester NHS Trust, Leicester, UK; 5 Division of Cardiovascular Medicine, Radcliffe Department of Medicine, Oxford Centre for Clinical Magnetic Resonance Research, National Institute for Health Research Oxford Biomedical Research Centre, Oxford University Hospitals NHS Foundation Trust, John Radcliffe Hospital, University of Oxford, Oxford, UK; 6 POLARIS Imaging Group, The Department of Infection, Immunity and Cardiovascular Disease, The University of Sheffield Faculty of Medicine Dentistry and Health, Sheffield, UK; 7 Insigneo Institute for in silico Medicine, The University of Sheffield Faculty of Medicine Dentistry and Health, Sheffield, UK; 8 Oxford Centre for Functional MRI of the Brain, Wellcome Centre for Integrative Neuroimaging, Nuffield Department of Clinical Neurosciences, University of Oxford, Oxford, UK; 9 Sir Peter Mansfield Imaging Centre, School of Physics and Astronomy, University of Nottingham, Nottingham, UK; 10 National Institute for Health Research Nottingham Biomedical Research Centre, University of Nottingham, Nottingham, UK; 11 National Institute for Health Research Cambridge Clinical Research Facility and Biomedical Research Centre, University of Cambridge, Cambridge, UK; 12 Institute of Cardiovascular and Medical Sciences and British Heart Foundation Glasgow Cardiovascular Research Centre, University of Glasgow, Glasgow, UK; 13 Diabetes Research Centre, University of Leicester, Leicester, UK; 14 Leicester National Institute for Health Research Biomedical Research Centre (Respiratory theme), Leicester, UK; 15 Infection, Inflammation and Immunity, University of Leicester, Leicester, UK; 16 Baker Heart and Diabetes Institute South Australia, Melbourne, Victoria, Australia

**Keywords:** COVID-19, Post-Acute COVID-19 Syndrome, Magnetic Resonance Imaging, Cardiovascular imaging, SARS-CoV-2 Infection, Surveys and Questionnaires

## Abstract

**Introduction:**

SARS-CoV-2 disease (COVID-19) has had an enormous health and economic impact globally. Although primarily a respiratory illness, multi-organ involvement is common in COVID-19, with evidence of vascular-mediated damage in the heart, liver, kidneys and brain in a substantial proportion of patients following moderate-to-severe infection. The pathophysiology and long-term clinical implications of multi-organ injury remain to be fully elucidated. Age, gender, ethnicity, frailty and deprivation are key determinants of infection severity, and both morbidity and mortality appear higher in patients with underlying comorbidities such as ischaemic heart disease, hypertension and diabetes. Our aim is to gain mechanistic insights into the pathophysiology of multiorgan dysfunction in people with COVID-19 and maximise the impact of national COVID-19 studies with a comparison group of COVID-negative controls.

**Methods and analysis:**

COmorbidities and Sociodemographic factors on Multiorgan Injury following COVID-19 (COSMIC) is a prospective, multicentre UK study which will recruit 200 subjects without clinical evidence of prior COVID-19 and perform extensive phenotyping with multiorgan imaging, biobank serum storage, functional assessment and patient reported outcome measures, providing a robust control population to facilitate current work and serve as an invaluable bioresource for future observational studies.

**Ethics and dissemination:**

Approved by the National Research Ethics Service Committee East Midlands (REC reference 19/EM/0295). Results will be disseminated via peer-reviewed journals and scientific meetings.

**Trial registration number:**

COSMIC is registered as an extension of C-MORE (Capturing Multi-ORgan Effects of COVID-19) on ClinicalTrials.gov (NCT04510025).

**STRENGTHS AND LIMITATIONS OF THIS STUDY**

- Prospective, longitudinal study of COVID-19 negative controls with diverse ethnic, sociodemographic and comorbidity profiles
- Extensive phenotyping of COVID-19 negative individuals with comprehensive multiorgan, vascular and lung imaging and self-reported measures of quality of life, frailty, cognitive function, mental health and functional capacity
- Blinded MRI with core laboratory analysis
- Use of nucleocapsid-specific COVID antibody assay to exclude previous infection

## Background

 COVID-19 has had a devastating global impact with lasting health and economic consequences. Declared as a pandemic on 11 March 2020, to date, over 770 million cases and 7 million fatalities have been attributed to the disease globally,^1^ making COVID-19 the most severe pandemic of the last century. Early initiatives through concerted global research efforts led to the use of immunomodulatory therapies and the development of vaccines to reduce disease severity and risk of mortality.^2^,^7^ However, a syndrome of persistent physical and neuropsychiatric symptoms became increasingly recognised among survivors,^8 9^ leading to the genesis of the term ‘long COVID’—defined by the persistence of symptoms beyond 12 weeks of infection.^10^ Long COVID has been shown to affect a significant proportion of non-hospitalised (10–30%), hospitalised (50–70%) and vaccinated (10–12%) cases.^11 12^ Although the mechanisms underlying long COVID are still poorly understood, a complex interplay of factors affecting multiorgan health including direct viral cytopathic effects,^13^ immune response dysregulation,^14^ coagulatory dysfunction,^15^,^17^ persistent inflammation,^18^ endothelial injury^19^ and disruption to the renin–angiotensin–aldosterone system^20^ have been postulated to contribute to the prolonged disabling syndrome.

### Multiorgan involvement in COVID-19

At an organ level, there are now several studies which describe the extent of COVID-19-mediated injury to vital organs both in the acute and early postacute phase. From a cardiac perspective, numerous studies have shown that the risks of myocarditis, heart failure, arrhythmias and thrombosis are significantly increased during the acute phase of infection^21^,^24^ and are thought to persist into the recovery phase.^25^ Troponin elevation indicates myocardial injury^26 27^ and has been associated with significant mortality,^28 29^ with additional prognostication from the degree of elevation.^30^ Cardiac magnetic resonance imaging (MRI) in the acute and postacute setting has revealed both ischaemic and non-ischaemic scarring.^31^,^33^ The neurological manifestations of COVID-19 range from mild symptoms such as headache, ageusia and dizziness^34^ to severe and incapacitating complications such as encephalopathy^35 36^ and strokes,^37 38^ leading to prolonged hospitalisation and mortality. Long-term, patients may suffer cognitive impairment, fatigue and other persistent symptoms,^39^,^41^ necessitating continued research into these effects in order to develop comprehensive care strategies for affected individuals. Hepatic manifestations in acute COVID-19 may range from mild elevations in liver enzymes indicative of acute liver injury to severe hepatic failure,^42 43^ characterised by elevated aspartate aminotransferase and alanine aminotransaminase levels indicating hepatocellular injury. This may be accompanied by elevated bilirubin and alkaline phosphatase, indicating cholestasis.^44 45^ Acute liver injury correlates with greater disease severity,^46^ ventilatory requirement^47^ and higher mortality,^48^,^51^ though persistent hepatic dysfunction postrecovery is rare.^52^ COVID-19 may also lead to acute kidney injury, the extent of which varies widely among hospitalised patients^53 54^ and significantly increases mortality risk.^55^,^57^ Common causes include renal hypoperfusion and multiorgan dysfunction, with severe cases often showing rapid decline due to conditions such as collapsing glomerulopathy.^58 59^ Long-term renal sequelae may arise from persistent inflammation and ischaemic damage despite improvement in biochemical markers of renal function.

These observations have spurred numerous prospective cardiac and multiorgan imaging studies across the UK, such as C-MORE (Capturing Multi-ORgan Effects of COVID-19),^60^ COVID-HEART,^33^ CISCO-19 (Cardiac Imaging in SARS COronavirus disease-19)^61^ and PHOSP-COVID (Post-HOSPitalisation COVID-19),^62^ aimed at understanding the medium- to long-term impacts of COVID-19 on multiorgan health. Despite these efforts, many studies face limitations due to the lack of robust control groups that are accurately matched for age, sex and comorbidities with the post-COVID population. This is crucial as conditions such as hypertension, diabetes and cardiovascular diseases may significantly affect multiorgan function and influence the severity of COVID-19^63^,^66^ as well as the risk of long COVID.^8^ Moreover, imaging performed postinfection may fail to discriminate whether organ damage is directly due to COVID-19 or pre-existing comorbidities.

### Risk factors for infection severity in COVID-19

Age is another critical factor determining the severity of COVID-19, with older adults facing significantly higher risk of COVID-19 and higher mortality rates^67 68^ owing to the presence of pre-existing chronic conditions. Gender disparities are also apparent, with males generally experiencing more severe outcomes and higher mortality,^67^,^69^ whereas females report more persistent symptoms.^70^ Obesity is also a significant predictor of severe COVID-19 outcomes^63 71 72^ and contributes to persistent post-COVID manifestations, while also independently affecting multiorgan health. Ethnic disparities are also evident, with Hispanic and Black people in the USA^73 74^ and ethnic minorities in the UK^75^ facing higher rates of hospital admission and mortality. These observations likely reflect socioeconomic, housing and occupational factors as well as associated comorbidities. Importantly, these factors may adversely affect multiorgan health and recovery independent of COVID-19 exposure.

### Summary

Given the close link between sociodemographic factors, health status and COVID-19 severity, there is a serious unmet need for well-controlled, demographically matched studies to understand the long-term impact of COVID-19 on organ health. The COSMIC study (assessing the impact of COmorbidities and Sociodemographic factors on Multiorgan Injury following COVID-19) is designed to address this gap and seeks to assess cardiac and multiorgan dysfunction in COVID-19 negative controls and will evaluate the specific effects of sociodemographic factors and comorbidities on organ health.

### Aims and objectives

To examine the prevalence of cardiac and multiorgan damage in non-COVID controls, establishing a foundational dataset to examine the impact of sociodemographic and comorbidity factors on broader health outcomes such as quality of life, cognitive function and mental health in a non-COVID-19 population. The COSMIC cohort will then serve as a comparator group for other national COVID-19 studies including C-MORE^60^ and COVID-HEART,^33^ with aligned imaging and clinical protocols, providing critical control data to enhance understanding of the broader impacts of the disease.

## Methods

### Study design

Prospective, multicentre, longitudinal study of UK COVID-19 negative controls with a diverse range of ethnic, sociodemographic and comorbidity profiles.

#### Primary outcome measure

- Prevalence of cardiac injury in COVID-19 negative controls.

#### Secondary outcome measures

- Prevalence of multiorgan dysfunction in COVID-19 negative controls.
- Prevalence of microvascular and macrovascular abnormalities in COVID-19 negative controls.

#### Exploratory outcome measures

- Patient reported outcome measures (PROMs) including self-reported quality of life, level of frailty, cognitive function, mental health and functional capacity.

### Study population

#### Inclusion criteria

- Adults (≥18 years) with no history of previous COVID-19 and negative serology (as determined by nucleocapsid antibody testing).
- Willingness to share pseudonymised data, images and blood samples with other ethically approved COVID-19 studies.

#### Exclusion criteria

- Current or previous COVID-19 or symptoms suggestive of COVID-19 and/or positive COVID-19 antigen testing.
- Absolute contraindications to MRI or gadolinium-based contrast agents (eg, severe claustrophobia, pregnancy, breast-feeding, ferromagnetic implants, inability to lie flat, severe renal impairment with estimated glomerular filtration rate <30 mL/min/m^2^).

### Study setting

Control participants will be recruited from regions served by four assessment centres (Leicester, Oxford, Leeds and Glasgow). All centres will have access to 3 Tesla MRI scanning capabilities, with subjects undergoing a single study visit.

### Screening and recruitment

**Figure 1:**
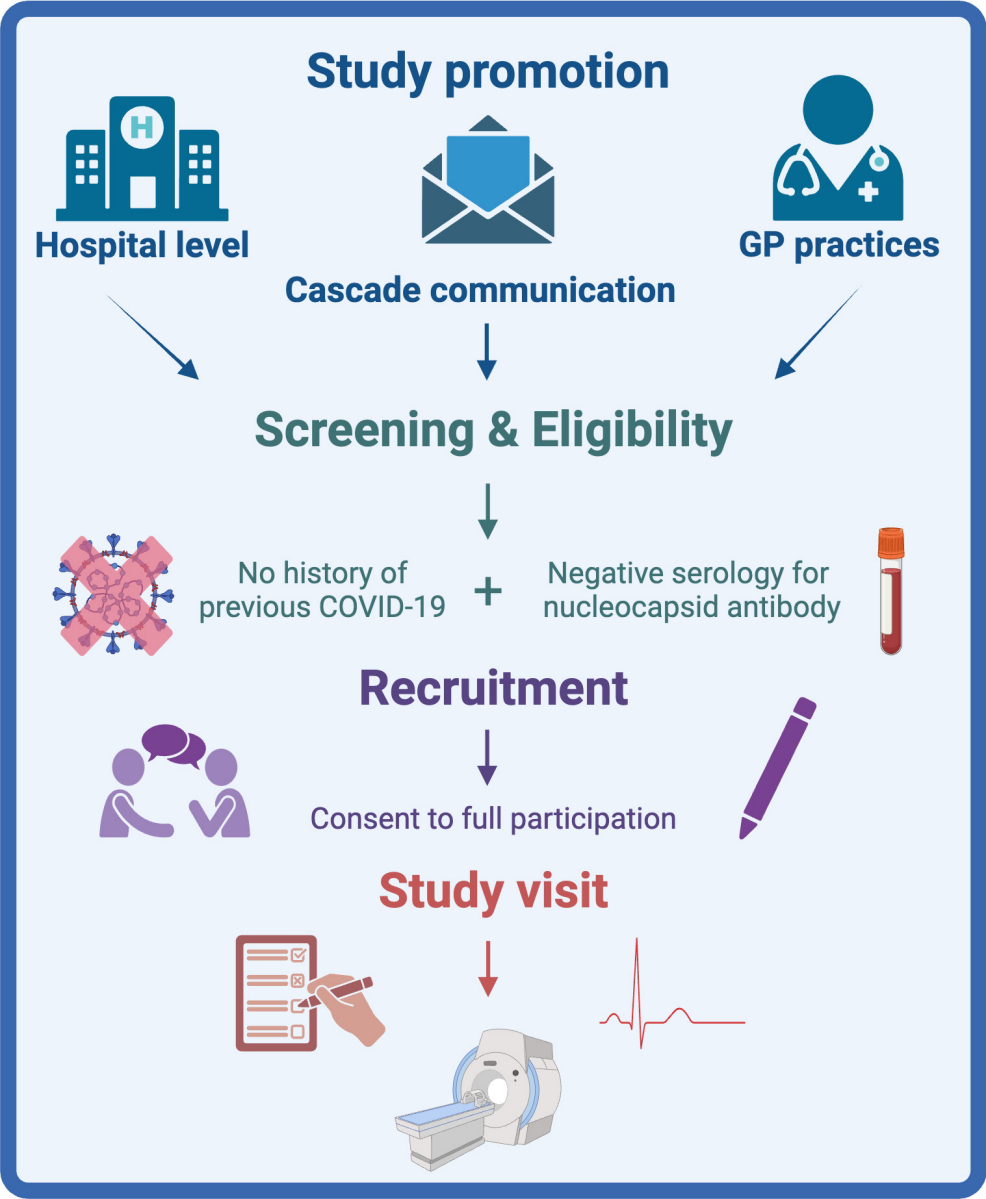
COSMIC screening and recruitment flow diagram. GP, general practitioner.

The study will be promoted at an individual hospital level through posters, social media and work with local general practices to identify suitable subjects (figure 1). Additionally, cascade communication to participants of other studies who have provided consent to be invited to other relevant studies will be utilised. The consent process is comprised of two stages: consent to screening and consent to full participation. Potential participants will be invited to attend a screening and eligibility visit at which they will be asked a series of screening questions about their health and confirm the absence of a history of possible previous COVID-19. All participants screened for inclusion will require a blood sample for viral serology to identify previous COVID-19 (nucleocapsid antibody). If negative, the subject will be eligible for enrolment. Eligible participants will provide written informed consent as per the standards of Good Clinical Practice and before any study procedures are performed. Eligible participants will be invited to attend one of the assessment centres to participate in the remainder of the study assessments.

### Data collection

Source data will include hospital records, clinical charts, laboratory data, scan images and clinical correspondence.

### Patient and public involvement

The design of the study and participant-facing written materials (information leaflet and consent form) were reviewed and approved by the Leicester Biomedical Research Centre (BRC) Patient Public Involvement Group who additionally gave feedback on the conduct of the study visits.

### Medical history

A comprehensive medical, medication and social history will be obtained from each participant. The postcode-based index of multiple deprivation will be used as a marker of socioeconomic status.

### Investigations

**Figure 2:**
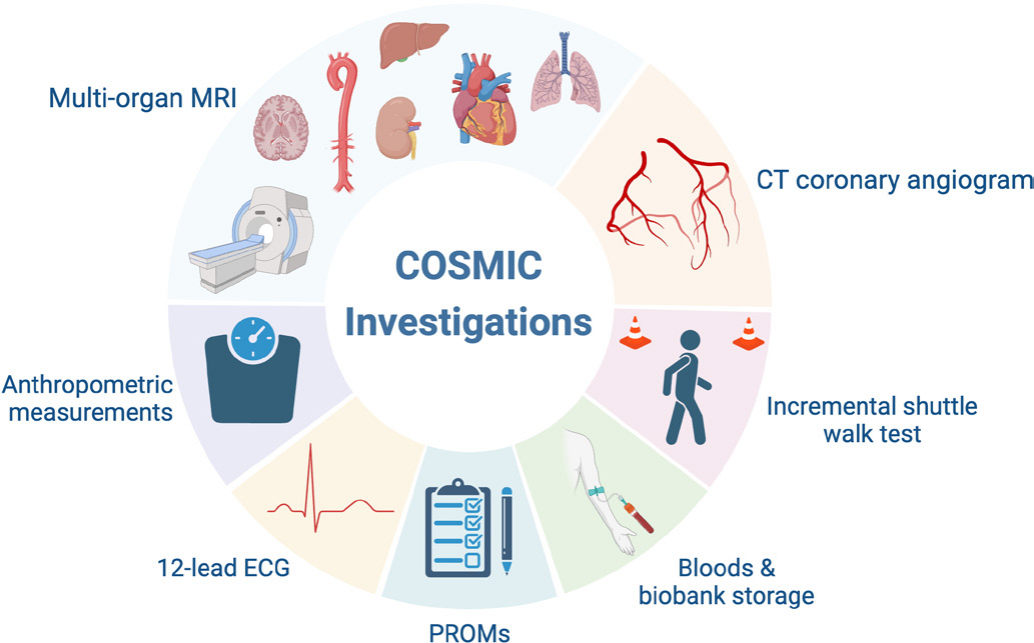
COSMIC investigations. PROMs, patient-reported outcome measures.

All investigations will be carried out in a single hospital visit (~4 hours). Participating subjects will be offered blood sampling for clinical laboratory tests and biobank storage, a 12-lead electrocardiogram (ECG), functional assessment, validated PROM questionnaires (see section PROM questionnaires), multiorgan MRI imaging and computed tomography coronary angiography (CTCA) (figure 2).

### Electrocardiogram

A 12-lead ECG will be performed prior to the MRI examination and reviewed by an appropriately trained healthcare professional.

### Laboratory investigations

Enrolled subjects will have blood samples taken for full blood count, urea and electrolytes, liver function, high-sensitivity C-reactive protein, haemoglobin A1c, high-sensitivity troponin I and B-type natriuretic peptide. Additionally, participants will undergo sample collection for biobank storage with plasma, serum and DNA extracted and frozen locally in freezers at −80°C. All samples will be transferred and stored at the British Heart Foundation Leicester Clinical Research Centre for subsequent whole genome/RNA sequencing and flow cytometry (subject to future funding). Blood samples for biomarker storage will be collected in 1×4.3 mL citrate tube, 1×9 mL serum separating tube (SST) and 1×9 mL tripotassium ethylenediaminetetraacetic acid (K3-EDTA) tube.

### PROM questionnaires

Study participants will be asked to complete patient-reported health status questionnaires at baseline to provide insight into self-reported quality of life, level of frailty, cognitive function, mental health and functional capacity. All PROMs are aligned with PHOSP-COVID and previously described.^62^

- Patient Health Questionnaire (PHQ-9).
- General Anxiety Disorder Questionnaire (GAD-7).
- Medical Research Council (MRC) Dyspnoea Scale.
- Montreal Cognitive Assessment (MoCA) Tool.
- Dyspnoea-12 score.
- Rockwood Clinical Frailty Scale (CFS).
- Functional Assessment of Chronic Illness Therapy-Fatigue (FACIT-F).
- EuroQol(EQ5D-5L).
- Strength, Assistance with Walking and Rising from a Chair, Climbing Stairs and Falls (SARC-F).
- General Practice Physical Activity Questionnaire (GPPAQ).
- Post-Traumatic Stress Disorder Checklist for DSM-5 (PCL-5).
- Brief Pain Inventory (BPI).
- Nottingham Extended Activities of Daily Living (NEADL).
- Pittsburgh Sleep Quality Index (PSQI).
- Morningness-Eveningness Questionnaire (MEQ).

### Functional assessment

The incremental shuttle walk test^76^ will be performed to assess maximal physical performance. The test will be conducted indoors with two cones set 9 m apart with subjects asked to walk around the cones in time to a set of auditory beeps. Walking speed progressively increases in 1 min intervals, with the participant walking for as long as they can until they are too breathless to continue, or can no longer keep pace with the auditory beeps.

### Magnetic resonance imaging

**Figure 3:**
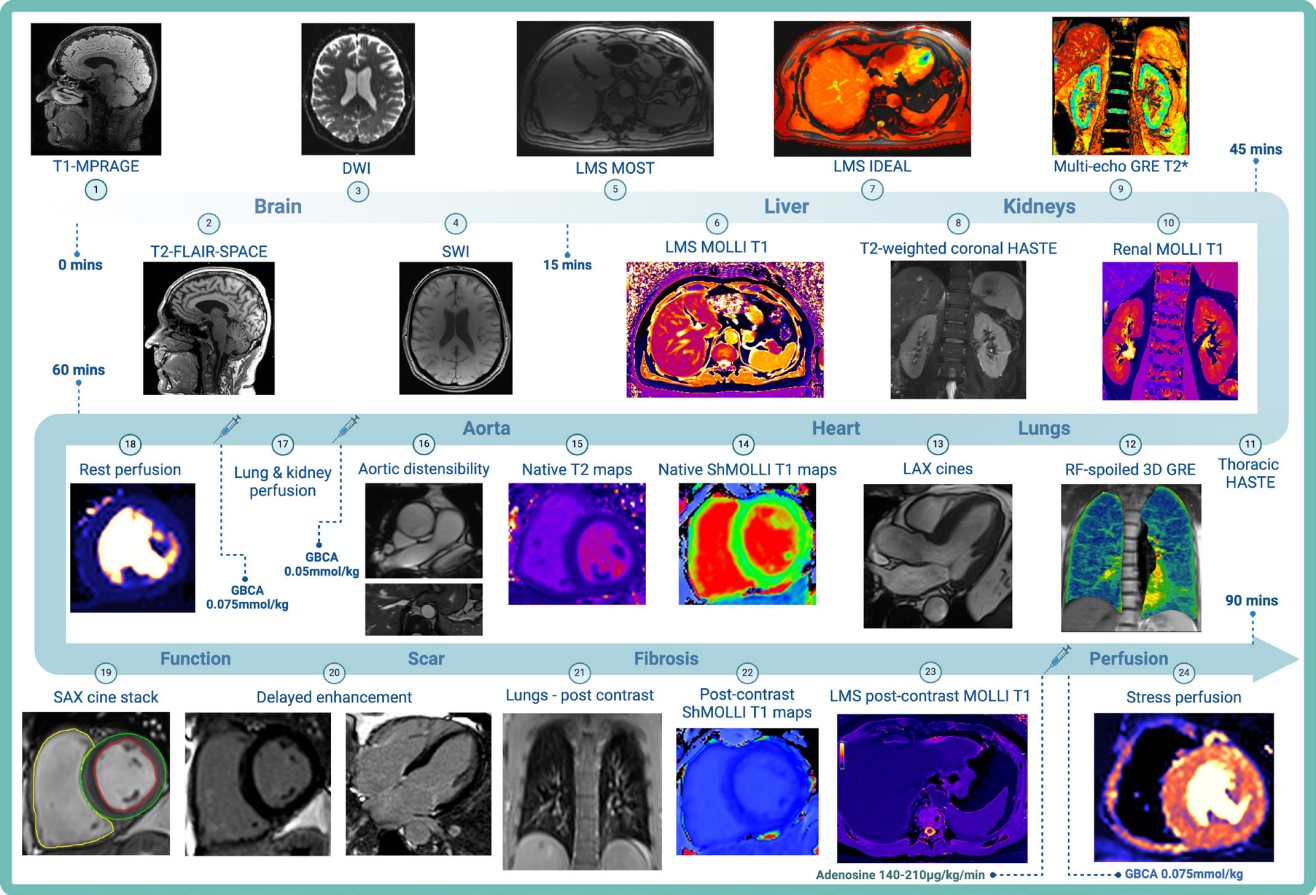
COSMIC multiorgan MRI protocol. DWI, diffusion-weighted imaging; GBCA, gadolinium-based contrast agent; GRE, gradient echo; HASTE, half-Fourier acquisition single-shot turbo spin echo; IDEAL, iterative decomposition of water fat with echo asymmetry and least-squares estimation; LAX, long axis; LMS, liver multi-scan; MOLLI, modified Look-Locker inversion recovery; MOST, magnitude only thin-slice T2*; MPRAGE, magnetisation-prepared rapid acquisition with gradient echo; RF, radiofrequency; SAX, short axis; ShMOLLI, shortened modified Look-Locker inversion recovery; SWI, susceptibility-weighted imaging; T2-FLAIR, T2-weighted fluid-attenuated inversion recovery; 3D, three-dimensional.

The MRI protocol (figure 3) includes the acquisition of multiorgan (brain, lungs, liver and kidneys) (online supplemental table 1) and cardiac imaging (online supplemental table 2) on a 3 Tesla Siemens (Erlangen, Germany) scanner (Prisma—Oxford, Leeds, Glasgow and Skyra—Leicester) aligned with the C-MORE study.^60^ The MRI protocol will take approximately 90 min to complete, with a comprehensive assessment of lung parenchymal changes and perfusion defects, cerebral volumes and degree of neuroinflammation, ischaemia and haemorrhagic injury, hepatic iron content, fibroinflammation and steatosis, renal volumes, fibroinflammatory changes and corticomedullary differentiation. Accepted gadolinium-based contrast agents include gadoterate-based agents (eg, Dotarem and Clariscan) with an administration rate of 4 mL/s followed by a 20 mL 0.9% saline flush at 4 mL/s via a power injector.

### Cardiac magnetic resonance protocol

The cardiac MRI protocol will evaluate ventricular volumes and function, characterise patterns of myocardial inflammation and fibrosis and assess aortic distensibility. Optional adenosine stress perfusion imaging will be performed on willing participants. All cardiac imaging will use retrospective ECG gating unless arrhythmias are present, in which case prospective gating will be used. To decrease the breath-hold duration, parallel imaging will be used in all acquisitions. In participants with poor breath-holding, the spatial resolution will be decreased, and free-breathing (increasing the averages to three for cine imaging) or real-time acquisitions will be performed.

### Multiorgan abnormalities

Multiorgan abnormalities are defined by the presence of MRI abnormalities involving two or more organs:

- Brain—abnormal volumes or presence of inflammatory, ischaemic or haemorrhagic injury.
- Lungs—parenchymal or perfusion abnormalities.
- Liver—abnormal steatosis, inflammation, fibrosis or iron content.
- Kidneys—abnormal volumes, structural integrity or an excess of inflammation or fibrosis.
- Heart—impaired ventricular systolic function, evidence of inflammation, diffuse or focal fibrosis.

### Image analysis

Image analysis protocols and software will be identical to those used for the C-MORE study as previously described.^77^ Additionally, an automated inline quantitative stress perfusion^78^ and aortic distensibility sequence will be acquired and analysed as previously described.^79 80^ All scans will be anonymised with a unique code and analysed in core laboratories (cardiac—Leicester, Oxford; brain and liver—Oxford; kidneys—Nottingham; lungs—Sheffield). Readers will be blinded to all participant information (including comorbidities and case status). Relevant clinical findings will be reported to the responsible clinician who will inform the participant and the general practitioner if any changes to clinical care are required.

### Computed tomography

A calcium score and CTCA will be offered to participants to allow the assessment of the epicardial coronary arteries, perivascular inflammation (fat attenuation index) and lung parenchyma. CT imaging will be performed on a 128-slice dual-source scanner (at a minimum). All CT imaging will be prospectively acquired (single-phase acquisition) and ECG gated with a dose constraint of 400 mGy. The fat attenuation index will be analysed as previously described.^81^ Local radiologists at the respective assessment centres will analyse and report the CTCA and any extracardiac findings.

### Coronary calcium score

A coronary calcium score will be acquired during an inspiratory breath hold from the carina to the base of the heart. Typical cardiac reconstructions: axial, slice thickness 3 mm and slice interval 1.5 mm. Lung reconstructions: axial, slice thickness 2 mm and slice interval 2 mm.

### Computed tomography coronary angiography

Prior to the CTCA, participants will receive glyceryl trinitrate (1000 µg tablet sublingually or 800 µg via a sublingual pump) to promote coronary vasodilation. If required, intravenous beta-blockers (metoprolol) will be administered to achieve a target heart rate ≤65 beats per minute. The CTCA will be planned from the calcium score with six slices taken above the origin of the coronary tree and below their termination at the base of the heart. A test bolus will determine peak aortic opacification with 5 s added to ensure adequate coronary artery opacification. Sequence performed during an inspiratory breath hold with 60 mL of IV contrast followed by 40 mL of 50% 0.9% saline and 50% intravenous contrast. Typical sequence parameters: kVp 120 (dependent on patient body mass index), kV 70 (dependent on coronary calcium burden), slice thickness 0.75 mm, slice interval 0.5 mm and rotation time 0.28 s.

### Follow-up

Clinical outcome data will be collected up to 10 years following the study visit from electronic patient records.

### Study timetable

Ethics and regulatory approval were secured in January 2022. Study enrolment started in May 2022, and recruitment is expected to be completed in June 2024. A further 12 months will be allowed for data analysis, statistical analysis, manuscript preparation and final report.

### Statistical analysis

A specific database mirroring baseline assessments for C-MORE/PHOSP-COVID will be created in the validated and online REDCap (Research Electronic Database Capture) system by Information Technology specialists at the National Institute for Health Research Leicester BRC. After cleaning and locking, data will be summarised using standardised descriptive statistics. Analysis will be undertaken by an experienced medical statistician employed by the Leicester BRC. The primary and secondary endpoints will be assessed using an analysis of covariance adjusting for any residual differences between cases and controls for the presence of diabetes, body mass index, age, ethnicity, sex, coronary artery disease, hypertension and socioeconomic status.

### Power calculation

As this is an exploratory study to develop control groups for national post-COVID imaging studies and emerging postinfectious cohorts, power calculation depends on the cohorts being compared. A sample size of 176 subjects will afford 90% power (α significance 5%) to show an increase in the prevalence of cardiac abnormalities based on an estimated 10% prevalence in the control group and 20% in the COVID-positive group, from C-MORE pilot data^77^ and a study examining hospitalised patients.^82^ To account for a 10% dropout rate due to missing or incomplete data, 200 participants will be recruited.

## Discussion

The proposed COSMIC study is of direct clinical relevance to individuals at risk of COVID-19 infection or experiencing post-COVID manifestations. As a control group, it will afford mechanistic insights into the pathophysiology and prognostic implications of multiorgan injury following COVID-19 and potentially following hospitalisation for other novel viral infections. Recent multiorgan imaging studies in post-COVID patients such as C-MORE,^60^ COVER-SCAN (mapping organ health in recovery from COVID-19 disease due to SARS-CoV-2 infection)^83^ and COVID-HEART^33^ have limitations including younger control groups with fewer comorbidities, absence of contrast-enhanced imaging to detect fibrosis/perfusion (eg, in COVER-SCAN) and lack of ethnically and sociodemographically matched controls. The COSMIC study provides a robust, contemporary uninfected control group that will be matched to the post-COVID cohorts for age, comorbidities and sociodemographic factors, with comprehensive multiorgan, vascular and lung imaging. However, challenges are likely to be encountered in recruiting participants with a wide range of comorbidity profiles given the limited pool of subjects without a history of prior COVID-19. Although the presence of nucleocapsid-specific antibodies will be utilised as a marker for previous infection, false negative results may arise from antibody waning. In this study, nucleocapsid antibody testing will be used in conjunction with patient-reported history of COVID-19 and prior antigen testing, but previous asymptomatic infection cannot definitively be excluded.

Importantly, the comprehensive clinical assessment of COVID-19 negative controls through a series of questionnaires evaluating self-reported quality of life, level of frailty, cognitive function, mental health and functional capacity paired with deep imaging phenotyping is unique. The parallel assessment of patients using the PHQ-9, GAD-7, MRC Dyspnoea Scale, MoCA and many others will also provide a control group for UK-wide post-COVID follow-up studies such as PHOSP-COVID—a large multicentre study of 8000 previously hospitalised individuals, currently lacking a well-matched control group. Additionally, COSMIC is collecting data on CT lung and vascular inflammation, thus serving as a control to other studies including C19-RS (COVID-19 radiotranscriptomic signature), ORFAN (The Oxford risk factors and non-invasive imaging study)^84^ and the PHOSP-COVID lung fibrosis working group^85^ which focus on the impact of COVID-19 on vascular inflammation, thrombosis and pulmonary interstitial lung disease.

This meticulous approach will enable improved risk stratification and elucidation of mechanistic drivers contributing to severe disease across diverse populations. For patients with long COVID, this work may promote a better understanding of the mechanisms of persistent symptoms and impaired quality of life. Furthermore, the extensive characterisation and phenotyping of a large cohort of COVID-negative controls, coupled with comprehensive multiorgan imaging and serum storage, will provide an invaluable bioresource to support future research into complex interactions between sociodemographic factors, comorbidities and multiorgan health. Despite the success of the vaccine programme, immune escape and viral resistance to antibody responses (following natural infection or vaccination) are expected to occur due to selection pressures on the virus, and thus the likelihood that COVID-19 will be cured remains low. Consequently, insights gained from the COSMIC study into both acute illness and long COVID will enable better preinfection targeting of preventive measures and individualised therapy in infected patients during potential future pandemics.

## Ethics and dissemination

The study was approved by the National Research Ethics Service Committee East Midlands (REC reference 19/EM/0295) and conducted according to the declaration of Helsinki. COSMIC is registered as an extension of C-MORE on ClinicalTrials.gov (NCT04510025). Trial findings will be published in peer-reviewed journals and disseminated at international conferences and scientific meetings.

## supplementary material

10.1136/bmjopen-2024-089508 online supplemental file 1

10.1136/bmjopen-2024-089508 online supplemental file 2
