# Supplementary material for: Assessing the impact of COmorbidities and Sociodemographic factors on Multiorgan Injury following COVID-19: rationale and protocol design of COSMIC, a UK multicentre observational study of COVID-negative controls
Source: BMJ Open. 2025 Mar 6;15(3):e089508. doi: 10.1136/bmjopen-2024-089508 (PMC11887317; doi:10.1136/bmjopen-2024-089508)
Supplement: online supplemental file 1 [file bmjopen-15-3-s001.docx]

**Supplementary table 1:** Multi-organ magnetic resonance imaging protocol

| **Sequence** | **Typical sequence parameters** |
| --- | --- |
| Brain | |
| High-resolution T1-weighted magnetisation prepared rapid acquisition with gradient echo (MPRAGE) | TR/TE/TI 2000/1.97/880ms. Acceleration factor 2. Flip angle 8°. FOV 256mm. FOV phase 100%. Matrix 256x256x208. Voxel dimension 1x1x1mm. |
| T2-weighted fluid attenuated inversion recovery  (T2-FLAIR-SPACE) | TR/TE/TI 5000/386/1800ms. Acceleration factor 3. FOV 256mm. FOV phase 100%. Matrix 256x256x192. Voxel dimension 1x1x1.05mm. |
| Diffusion-weighted imaging (DWI) | TR/TE 10700/78ms. Flip angle 90°. FOV 230mm. FOV phase 100%. Matrix 104x104x72. Voxel dimension 2x2x2mm. 6/8 Partial Fourier, b=0, 1000s/mm2, 3 sequentially applied diffusion gradient directions (x, y, z), plus blip-reversed b=0* |
| Susceptibility-weighted imaging (3D-SWI) | TR/TE_1_/TE_2_ 27/9.42/20ms. Acceleration factor 2. Flip angle 15°. FOV 230mm. FOV phase 91%. Matrix 232x256x48. Voxel dimension 0.9x0.9x3mm. |
| Lungs | |
| Free breathing thoracic axial half-Fourier acquisition single-shot turbo spin echo (HASTE) | TR/TE 750/49ms. Flip angle 160°. FOV 380mm. FOV phase 72%. Matrix 256x184. Slice thickness 8mm. Distance factor 0%. 35 slices. |
| Inspiratory and expiratory radio-frequency spoiled 3D coronal gradient echo (GRE) native and post contrast | TR/TE 1.87/0.67ms. Flip angle 3°. FOV 400mm. FOV phase 100%. Matrix 128x128. Voxel dimension 3.1x3.1x3mm. Slice thickness 3mm, Distance factor 20%. 88 slices. |
| Lungs and kidney perfusion  Dynamic contrast enhanced GRE  *Administration of 0.05mmol/kg GBCA* | TR/TE 1.47/0.5ms. Flip angle 17°. FOV 450mm. FOV phase 100%. Matrix 64x64. Voxel dimension 7x7x6.3mm. Slice thickness 6.3mm. Distance factor 20%. 40 slices/slab. 60 measurements. |
| Liver | |
| Liver multi scan (LMS)  magnitude only thin-slice T2* (MOST) | TR/TE/Echo spacing 12/1.3-9.84/1.2ms. Flip angle 9°. FOV 440mm. FOV phase 81%. Matrix 256x208. Slice thickness 3mm. |
| LMS modified look-locker inversion recovery (MOLLI)  T1 native and post-contrast map | TR/TE/initial TI 373/1.05/100ms. Flip angle 35°. FOV 440mm. FOV phase 75%. Matrix 382x288. Voxel dimension 2.3x2.3.8mm. Slice thickness 8mm. Distance factor 25%. |
| LMS iterative decomposition of water and fat with echo asymmetry and least-squares estimation (IDEAL) | TR/TE/Echo spacing 15/1.1-13.2/1.1ms. Flip angle 3°. FOV 440mm. FOV phase 91%. Matrix 256x232. Slice thickness 10mm. Distance factor 50%. |
| Kidneys | |
| T2-weighted coronal HASTE | TR/TE 438/61ms. GRAPPA factor 3. Refocus angle 120°. Bandwidth 781Hz  FOV 384mm. FOV phase 100%. Matrix 256x256. Voxel dimension 1.5x1.5x5mm. Slice thickness 5mm. |
| Multi-echo GRE oblique coronal T2* | TR/TE/Echo spacing 81/9.84-63.96/4.92ms. GRAPPA factor 3. Flip angle 25°  FOV 288mm. FOV phase 100%. Matrix 192x192. Voxel dimension 1.5x1.5x5mm. Slice thickness 5mm. |
| MOLLI T1 oblique coronal map | TR/TE/initial TI 274.3/1.15/100ms. Flip angle 35°. FOV 320mm. FOV phase 100%. Matrix 384x384. Voxel dimension 0.8x0.8x5.5mm. Slice thickness 5.5mm. |

^†^**Abbreviations**: *DWI* diffusion-weighted imaging, *FOV* field of view, *GBCA* gadolinium-based contrast agent, *GRAPPA* generalised autocalibrating partial parallel calibration, GRE gradient echo, HASTE half-Fourier single-shot turbo spin-echo, LMS liver multi scan, MOLLI modified look locker inversion recovery, *MPRAGE* magnetisation-prepared rapid acquisition with gradient echo, *SWI* susceptibility-weighted imaging, *TE* echo time, *TI* inversion time, *TR* repetition time, *T2-FLAIR* T2-weighted fluid attenuated inversion recovery, *3D* three dimensional.

*The diffusion sequence was kindly provided by the Centre for Magnetic Resonance Research, University of Minnesota, Minneapolis, Minnesota, USA.
