# Supplementary material for: Assessing the impact of COmorbidities and Sociodemographic factors on Multiorgan Injury following COVID-19: rationale and protocol design of COSMIC, a UK multicentre observational study of COVID-negative controls
Source: BMJ Open. 2025 Mar 6;15(3):e089508. doi: 10.1136/bmjopen-2024-089508 (PMC11887317; doi:10.1136/bmjopen-2024-089508)
Supplement: online supplemental file 2 [file bmjopen-15-3-s002.docx]

**Supplementary table 2:** Cardiac magnetic resonance imaging protocol

| **Sequence** | **Planning** | **Typical sequence parameters** |
| --- | --- | --- |
| Long axis (LAX) cines: 4, 2, 3-chamber. | Balanced steady-state free precession (bSSFP) sequence. Real-time imaging if poor breath-hold or arrhythmia. | TR/TE/Echo spacing 37.92/1.38/3.16ms. Flip angle 65°. FOV 380mm. FOV phase 84%. Matrix 208x174. Slice thickness 7mm. Distance factor 20%. |
| Native and post contrast T1 mapping: Basal, mid and apical left ventricle (LV) short axis (SAX). | Shortened modified Look-Locker inversion (ShMOLLI) sequence. Shimming tightly planned around the LV. Maps quality checked for artefact (all pixels in the heart and other organs should appear white on R^2^ map). Post contrast images acquired ≥10 minutes following GBCA with same sequence, parameters and slice positions. | TR/TE/initial TI 379/1.07/100ms. Flip angle 35°. FOV 360mm. FOV phase 75%. Matrix 288x384. Slice thickness 8mm. Distance factor 25%. |
| Native T2 mapping: Basal, mid and apical LV SAX. | T2-prepped bSSFP sequence (MyoMaps, Siemens). Slice location and adjustment volumes copied from T1 maps. | TR/TE/Echo spacing 222/1.3/3ms. Flip angle 20°. FOV 360mm. FOV phase 80%. Matrix 154x192. Slice thickness 8mm. Distance factor 25%. |
| Aortic distensibility: Level of the right pulmonary artery and diaphragm. | Axial bSSFP cine planned from HASTE and sagittal localiser to aid angulation through the aorta. Acquisition box shifted 12cm caudally to image descending aorta. Blood pressure measured prior to and following imaging. | TR/TE 43.5/1.26ms. Flip angle 55°. FOV 380mm. FOV phase 100%. Matrix 384x384. Slice thickness 6mm. Distance factor 20%. 100 phases acquired. |
| Optional rest and stress perfusion: Basal, mid and apical LV SAX.  *0.075mmol/kg of GBCA for rest and stress (total 0.15mmol/kg)* | Free-breathing pixel-wise perfusion mapping sequence.  Stress images acquired after satisfactory haemodynamic and/or symptomatic response to adenosine infusion at 140-210 micrograms/kg/minute for 3-6 minutes. | TR/TE 142.18/1.0ms Flip angle 14°. FOV 360mm. FOV phase 75%. Slice thickness 8mm.  60 measurements acquired for rest and stress. |
| SAX cine stack. | bSSFP sequence with parallel cines until the ventricles have been covered. Real time imaging can be performed if inadequate breath-holds or arrhythmia. | TR/TE 37.92/1.38ms. Flip angle 65°. FOV 380mm. FOV phase 84%. Matrix 208x174. Slice thickness 7mm. Distance factor 43%. |
| Inversion scout: 4-chamber LAX view. | Performed ≥5 minutes post contrast. | TR/TE 28.71/1.41ms. Flip angle 35°. FOV 340mm. FOV phase 81.4%. Matrix 192x78. Slice thickness 8mm. Distance factor 20%. |
| Late gadolinium enhancement: 4, 2, 3-chamber LAX and contiguous stack of LV SAX images. | Breath-hold segmented phase-sensitive inversion recovery sequence. Optional single-shot acquisitions if the participant tiring or inadequate breath-holds. | TR/TE/Echo spacing 700/1.19/2.90ms. Flip angle 55°. FOV 380mm. FOV phase 68.8%. Matrix 256x176 Slice thickness 7mm. Distance factor 43%. |

^†^**Abbreviations**: *bSSP* balanced steady-state free precession, *FOV* field of view, *GBCA* gadolinium-based contrast agent, *HASTE* half-Fourier single-shot turbo spin-echo, *LAX* long axis, *LV* left ventricle, *ShMOLLI* shortened modified look locker inversion recovery, *SAX* short axis, *TE* echo time, *TI* inversion time, *TR* repetition time.
