# Supplementary material for: Assessing the impact of COmorbidities and Sociodemographic factors on Multiorgan Injury following COVID-19: rationale and protocol design of COSMIC, a UK multicentre observational study of COVID-negative controls
Source: BMJ Open. 2025 Mar 6;15(3):e089508. doi: 10.1136/bmjopen-2024-089508 (PMC11887317; doi:10.1136/bmjopen-2024-089508)
Supplement: Supplementary file 3 [file bmjopen-2024-089508.reviewer_comments.pdf]

PEER REVIEW HISTORY

BMJ Open publishes all reviews undertaken for accepted manuscripts. Reviewers are asked to complete a checklist review form and are provided with free text boxes to elaborate on their assessment. These free text comments are reproduced below.

ARTICLE DETAILS

Title (Provisional)

Assessing the impact of COmorbidities and Sociodemographic factors on Multiorgan Injury following COVID-19: Rationale and protocol design of COSMIC, a UK multi-centre observational study of COVID-negative controls.

Authors

Shergill, Simran; Elshibly, Mohamed; Hothi, Sandeep S; Parke, Kelly S; England, Rachel J; Wormleighton, Joanne V; Hudson, George J; Tunnicliffe, Elizabeth M; Wild, James; Smith, Stephen M; Francis, Sue; Toshner, Mark; Sattar, Naveed; Khunti, Kamlesh; Brightling, Christopher E; Antoniades, Charalambos; Berry, Colin; Greenwood, John Pierre; Moss, Alastair; Neubauer, Stefan; McCann, Gerry P; Raman, Betty; Arnold, Jayanth Ranjit

---

VERSION 1 - REVIEW

**Reviewer**	**1**
**Name**	**Fornari, Carla**
**Affiliation**	**University of Milan–Bicocca, Medicine and Surgery**
**Date**	**09-Oct-2024**
**COI**	**None**

I think the topic of the article is very important for future research. Thank you for the great effort. The article is well written and all study details are clearly specified.

**Reviewer**	**2**
**Name**	**Batista, Sandro**
**Affiliation**	**UFG, Internal Medicine**
**Date**	**15-Jan-2025**
**COI**	**None**

Dear authors,

I am very pleased for the opportunity to review the manuscript draft - Study Protocol "Assessing the impact of COmorbidities and Sociodemographic factors on Multiorgan Injury following COVID-19: Rationale and protocol design of COSMIC, a UK multi-centre observational study of COVID-negative controls" submitted to BMJ Open, especially due to its potential for future innovation in health care delivery related to people suffering COVID-19.

It is a very interesting work and, for me, will bring crucial insights about long COVID. The study shows a comprehensive analysis of multiple variables, especially those related to PROMS and socioeconomic points. The study has a huge scientific potential especially as a comparator group for other national COVID-19 studies.

The authors followed all instructions for submitting the article. Ethical issues were adequately addressed.

Best regards,
